# High Expression of miR-204 in Chicken Atrophic Ovaries Promotes Granulosa Cell Apoptosis and Inhibits Autophagy

**DOI:** 10.3389/fcell.2020.580072

**Published:** 2020-11-05

**Authors:** Zhifu Cui, Lingbin Liu, Felix Kwame Amevor, Qing Zhu, Yan Wang, Diyan Li, Gang Shu, Yaofu Tian, Xiaoling Zhao

**Affiliations:** ^1^Farm Animal Genetic Resources Exploration and Innovation Key Laboratory of Sichuan Province, Sichuan Agricultural University, Chengdu, China; ^2^College of Animal Science and Technology, Southwest University, Chongqing, China; ^3^Department of Pharmacy, College of Veterinary Medicine, Sichuan Agricultural University, Chengdu, China

**Keywords:** chicken, atrophic ovary, miR-204, apoptosis, autophagy, signaling pathway

## Abstract

Chicken atrophic ovaries have decreased volume and are indicative of ovarian failure, presence of a tumor, or interrupted ovarian blood supply. Ovarian tumor is accompanied by an increase in follicular atresia, granulosa cell (GC) apoptosis, and autophagy. In a previous study, we found using high throughput sequencing that miR-204 is highly expressed in chicken atrophic ovaries. Thus, in the present study, we further investigated its function in GC apoptosis and autophagy. We found that overexpression of miR-204 reduced mRNA and protein levels of proliferation-related genes and increased apoptosis-related genes. Cell counting kit-8 (CCK-8), 5-ethynyl-2-deoxyuridine (EdU), and flow cytometry assays revealed that miR-204 inhibited GC proliferation and promoted apoptosis. Furthermore, we confirmed with reporter gene assays that *Forkhead box K2* (*FOXK2*) was directly targeted by miR-204. FOXK2, as a downstream regulator of phosphoinositide 3-kinase (PI3K)/AKT/mammalian target of rapamycin (mTOR) signal pathways, promoted GC proliferation and inhibited apoptosis. Subsequently, we observed that miR-204 was involved in GC autophagy by targeting *Transient Receptor Potential Melastatin 3* (*TRPM3*). The luciferase activities of the two binding sites of TRPM3 were decreased in response to treatment with a miR-204 mimic, and the autophagic flux was increased after miR-204 inhibition. However, overexpression of miR-204 had opposite results in autophagosomes and autolysosomes. miR-204 inhibits GC autophagy by suppressing the protein expression of TRPM3/AMP-activated protein kinase (AMPK)/ULK signaling pathway components. Inhibition of miR-204 enhanced autophagy by accumulating and degrading the protein levels of LC3-II (Microtubule Associated Protein Light Chain 3B) and p62 (Protein of 62 kDa), respectively, whereas miR-204 overexpression was associated with contrary results. Immunofluorescence staining showed that there was a significant reduction in the fluorescent intensity of LC3B, whereas p62 protein was increased after TRPM3 silencing. Collectively, our results indicate that miR-204 is highly expressed in chicken atrophic ovaries, which promotes GC apoptosis *via* repressing FOXK2 through the PI3K/AKT/mTOR pathway and inhibits autophagy by impeding the TRPM3/AMPK/ULK pathway.

## Introduction

The ovary plays vital roles in female reproductive performance (Castagna et al., 2004; Gethoffer et al., 2018; Zhao et al., 2018) by promoting ovulation (exocrine function) and hormone secretion (endocrine function) (Sirotkin et al., 2018; Zangirolamo et al., 2018; Russell, 2019). The ovary is an ideal model for the study of ovarian biology and follicular development (Bahr, 1991), and the follicular granulosa cells (GCs) are often regarded as an important marker of follicular development because of their unique structural characteristics and important role in follicular development (Johnson and Woods, 2009). The development and fate of follicles, maturation or atresia, depend on the state of GCs in the follicles. Proliferation of GCs promotes maturation of follicles, while apoptosis led to GC atresia (Matsudaminehata et al., 2006). Abnormal growth and differentiation of GCs cause ovarian diseases, such as premature ovarian failure (Shelling, 2010), polycystic ovarian syndrome (Jiang et al., 2015), and GC tumors (Pilsworth et al., 2018). Atrophic signifies a structure that is shrunken or diminished in size and function. An atrophic ovary has decreased ovarian tissue volume and is indicative of ovarian failure and interrupted ovarian blood supply, which may therefore affect reproduction in animals. In general, an atrophic ovary will not generate or produce healthy eggs and may therefore affect reproduction (Pilsworth et al., 2018).

MicroRNAs (miRNAs) are endogenous 22∼24-nucleotide-long, small non-coding, single-stranded RNAs (Kim, 2005), many of which regulate translation by binding to the 3′-untranslated regions (3′-UTRs) of their target mRNAs to inhibit protein translation (Lee et al., 1993; Bernstein et al., 2001; Bartel, 2004; Chekulaeva and Filipowicz, 2009). Several studies reported that miRNAs are involved in a variety of important cellular biological processes, including cell proliferation, differentiation, and apoptosis (Ambros, 2004; Valadi et al., 2007; Kosaka et al., 2010). In the ovary of different animals, many miRNAs participate in the entire process of ovarian follicle development, including follicle growth, atresia, ovulation, and regulating GC proliferation and apoptosis (Baley and Li, 2012; Kim et al., 2013; Imbar and Eisenberg, 2014). For instance, miR-224 regulated mouse GC proliferation (Yao et al., 2010), while miR-23a was differentially expressed in premature ovarian failure patients and was involved in GC apoptosis (Yang et al., 2012). Meanwhile, let-7 family members were preferentially expressed in the ovary, and let-7g promoted follicular GC apoptosis by targeting transforming growth factor-β1 (TGF-β1) (Zhou et al., 2015). miR-183-96-182 cluster regulates bovine GC proliferation by targeting FOXO1 (Gebremedhn et al., 2016); however, miR-10 family members repressed proliferation and induced apoptosis in ovarian GCs (Jiajie et al., 2017). Some miRNAs, such as miR-21 (Carletti et al., 2010), miR-15a (Sirotkin et al., 2014), miR-383 (Yin et al., 2014), miR-92a (Liu et al., 2014), miR-145 (Yan et al., 2012), and miR-34a (Tu et al., 2014), were reported to regulate GC growth. These findings demonstrate that miRNAs are expressed in the ovary and are actively involved in regulating animal reproduction.

Functions of miR-204 have been linked to many biological processes, including maintenance of joint homeostasis and protection against osteoarthritis (Huang et al., 2019), tumor growth (Mikhaylova et al., 2012), migration and anoikis of cancer cells (Sacconi et al., 2012; Zhang et al., 2013), and autophagy (Xiao et al., 2011; Hall et al., 2014). Recently, we performed a whole-transcriptome analysis of atrophic ovaries in broody chickens and found that miR-204 was differentially expressed between atrophic and normal ovaries (Liu et al., 2018), suggesting that miR-204 regulates ovarian function. Therefore, it is imperative to identify the regulators of miR-204 target genes and their roles in signaling pathways associated with both the development and function of the ovary. This should generate information that can be used for improving reproductive performance by controlling the key factors in the regulatory networks.

## Materials and Methods

### Animals and Sample Collection

Three hundred eighty-day-old chickens were raised at the Animal Breeding Farm, Sichuan Agricultural University (SAU) (Ya’an, China). Each of five egg-laying and broody birds was selected, and tissues were collected including hypothalamus, pituitary, heart, liver, spleen, lung, kidney, gizzard, glandular stomach, ovary, breast muscle, leg muscle, and small intestine. Atrophic ovaries were collected from broody birds and wrapped in foil, snap-frozen in liquid nitrogen, and transferred to a -80°C freezer for further analyses.

The animal experiments were approved by the Institutional Animal Care and Use Committee of Sichuan Agricultural University (Certification No. YCS-B2018102013). All experiments were conducted in accordance with the SAU Laboratory Animal Welfare and Ethics guidelines.

### Cell Culture and Transfection

All pre-ovulatory follicles were dissected from the ovary and placed in sterile Hank’s balanced salt solution. The GCs of follicles were isolated following the method of Gilbert (Gilbert et al., 1977), and the granular layers were digested using β-II collagenase (BaiTai Biotechnology, Chengdu, China). Cells were filtered with 200 mesh cell sieves and then resuspended in Dulbecco’s modified Eagle medium (DMEM) + 10% fetal bovine serum (Gibco, Grand Island, NY, United States) + 0.1% mixture of penicillin–streptomycin (Invitrogen, Carlsbad, CA, United States). GCs were isolated and cultured in the cell culture incubator at 37°C, 5% CO_2_, and 95% air saturated humidity for 3 h to ensure cell attachment. Thereafter, the medium was changed to remove non-adherent cells. All cells including GCs were further cultured in the cell culture incubator at 37°C, 5% CO_2_, and 95% air saturated humidity, which was followed by medium being changed every 24 h. Afterward, the transfection procedure was performed with *Forkhead box K2* (*FOXK2*) small interfering RNA (Si-FOXK2), *Transient Receptor Potential Melastatin 3* (*TRPM3*) small interfering RNA (Si-TRPM3), and a miR-204 mimic or inhibitor using lipofectamine 3000 reagent (Invitrogen, United States), according to the manufacturer’s directions. Oligonucleotide sequences are provided in Supplementary Table 1.

### Quantitative Real-Time PCR

Total RNA was extracted from the tissues and cells using TRIzol reagent (Takara, Tokyo, Japan) following the manufacturer’s instructions. Quantitative real-time PCR (qRT-PCR) analysis was conducted in reaction volumes of 15 μl containing 1.5 μl of cDNA, 0.3 μl of forward and reverse primers, 6.25 μl of TB Green^TM^ Premix (Takara), and 6.65 μl of DNase/RNase-Free Deionized Water (Tiangen, Beijing, China). Reaction conditions were based on the manufacturer’s instructions, and the 2^–ΔΔCt^ method was used to calculate fold changes in gene expression (Livak and Schmittgen, 2001). The β-actin and U6 genes were used as internal controls, and primer sequences are listed in Supplementary Table 2.

### Protein Extraction and Western Blot Analysis

Protein was extracted from the GCs using commercial protein extraction kits (BestBio Biotech Co., Ltd., Shanghai, China), and bicinchoninic acid kits (BestBio) were used to determine protein concentrations. The protein was denatured at 95°C for 5 min, and the total volume in each well included 16 μl of protein sample and 4 μl of reducing loading buffer (4:1). The protein was successively separated using 5% and 12% sodium dodecyl sulfate (SDS) polyacrylamide gel electrophoresis (Beyotime, Shanghai, China) and transferred to polyvinylidene difluoride membranes activated with methanol. Blocking buffer (Beyotime) was used to block the membranes at room temperature for 1 h, and the following primary antibodies were used to probe the target proteins: anti-FOXK2 [Cell Signaling Technology (CST), United States], cyclin-dependent kinase 2 (CDK2; ABclonal Technology, Wuhan, China), proliferating cell nuclear antigen (PCNA; ABclonal), Bcl-2 (Biorbyt, United Kingdom), caspase-3 (Abcam, Cambridge, United Kingdom), caspase-9 (Bioss, Beijing, China), phosphoinositide 3-kinase (PI3K; Bioss), Akt (CST), mammalian target of rapamycin (mTOR; Zen-Bio, Chengdu, China), TRPM3 (CST), AMP-activated protein kinase (AMPK; Bioss), ULK1 (Sigma Chemical Co., St. Louis, MO, United States), LC3 (CST), p62 (CST), and β-actin (Sigma), all overnight at 4°C. The membranes were rinsed with Wash Buffer (Beyotime), the corresponding secondary antibodies were added, and membranes were incubated for 1.5 h. The antibodies were diluted according to the manufacturer’s instructions.

### Cell Proliferation Assay

Primary GCs were cultured in 96-well plates. After transfection, cell proliferation was assessed with cell counting kit-8 (CCK-8; MeilunBio, Dalian, China) and a Cell-Light^TM^ 5-ethynyl-2-deoxyuridine (EdU) Kit (RiboBio, Guangzhou, China) according to the manufacturer’s protocols. Then, 10 μl of CCK-8 reagent was added to each well and incubated in the cell culture incubator for 2 h after which it was transfected at 12, 24, 36, and 48 h. The optical density (OD) value for each sample was detected with a microplate reader (Thermo Fisher, Varioskan LUX, United States) at 450 nm.

The state of proliferation of the GCs was determined using EdU kits adhering to the guidelines of the manufacturer. After addition of 100 μl of 50 μM EdU to each well, cells were incubated in the cell culture incubator for an additional 3 h. Cells were then washed with phosphate buffered saline (PBS) and then fixed with 4% paraformaldehyde for 30 min. Also, 50 μl of 2 mg/ml glycine was used to neutralize excess aldehyde groups, and 100 μl of 0.5% Triton X-100 PBS was added to increase the cell membrane permeability. Furthermore, 100 μl of Apollo was added, and the cells were subsequently incubated in a dark room at room temperature for 30 min. Afterward, the cells were washed with PBS, and the nucleus was stained with 100 ml of Hoechst 33342 reaction solutions, and then dark room temperature incubation continued for another 30 min. After the final incubation, a fluorescence microscope (DP80; Olympus, Japan) was used to visualize and quantify the numbers of EdU-stained cells. Three fields were randomly selected for statistical analysis.

### Cell Apoptosis Analysis

Cells were washed with PBS, and the concentration was adjusted to 10^6^ cells/ml. Also, 100 ml of single-cell suspension was centrifuged at 250 *g* for 5 min, and the supernatant solution was discarded, then 20 μl of binding buffer was added for cell resuspension. Subsequently, the cells were stained using 5 μl of Annexin V-FITC (Invitrogen, Australia) for 10 min, and then 10 μl of propidium iodide (PI; Invitrogen) was added for further staining in the dark at room temperature for 5 min. GC apoptosis was analyzed using flow cytometry (CytoFLEX, Backman, United States), and Kaluza 2.1 software was used to analyze the data.

### Dual-Luciferase Reporter Assay

DF-1 cell lines of chicken embryo fibroblast cells (DF-1 cells) were cultured with DMEM + 10% fetal bovine serum and seeded in 48-well plates. The cells were co-transfected with plasmid (*FOXK2* 3′UTR wild type or mutant type, *TRPM3* 3′UTR wild type or mutant type) and mimic negative control (NC) or miRNA mimic when cell density coverage reached 70∼80%. After 48 h, we detected luciferase activity using a luciferase reporter assay kit (Promega, Madison, WI, United States), according to the manufacturer’s instruction. The tests were conducted in triplicate.

### PI3K/AKT/mTOR Pathway Analysis

LY294002 (PI3K inhibitor) and 740Y-P (PI3K activator) were used to test the relationship between PI3K and FOXK2. LY294002 and 740Y-P were purchased from Selleck Chemicals (Houston, Texas, United States) and were preincubated with cells for 2 h (Tu et al., 2018). CCK-8 assay was used to determine the optimum concentration of LY294002 and 740Y-P treatments.

### Confocal Microscopy

The extent of an autophagic flux was evaluated with an adenovirus harboring tandem fluorescent mRFP-GFP-LC3 (Hanbio, Shanghai, China). Prior to the autophagic flux evaluation, the GCs were cultured on cell slides in six-well plates and transfected with miR-204 mimic and mimic NC or miR-204 inhibitor and inhibitor NC. The adenovirus was later added into the cells for 6–8 h following the manufacturer’s instructions. The culture medium was changed, and the GCs were further cultured for 48 h. Thereafter, the GCs were washed with cold PBS and later fixed with 4% paraformaldehyde for 30 min. The GCs were washed again for three consecutive times with PBS, after which they were observed under a confocal microscope (Olympus, Melville, NY, United States).

GCs were transfected with an interference vector (Si-*TRPM3*) and Si-NC. Later, the cells were washed with PBS for 5 min and were further fixed in 4% paraformaldehyde for 10 min. The cells were then washed with PBS, then 0.2% Triton X-100 was added to the cell, and incubation occurred for 10 min in order to ensure permeability of the cell membrane. The cells were washed and incubated with primary antibody rabbit anti-LC3B (CST) and mouse anti-p62 (CST) overnight at 4°C. Thereafter, the cells were washed three times and incubated with fluorescence-labeled secondary antibody in the dark at room temperature for 1 h. The cells were then washed three times in Tris-Buffered Saline Tween-20 (TBST), and then confocal microscopy was used to observe and analyze fluorescence intensity.

### Statistical Analysis

Data collected were subjected to statistical analyses using SPSS 20 Statistical software, and the mean of three replicates was evaluated and is displayed as mean ± standard error (SE). Significance was determined using Duncan’s multiple range tests and presented as *P* < 0.05 (^∗^) and *P* < 0.01 (^∗∗^).

## Results

### Bioinformatics Analysis and Target Gene Prediction of miR-204

**FIGURE 1:**
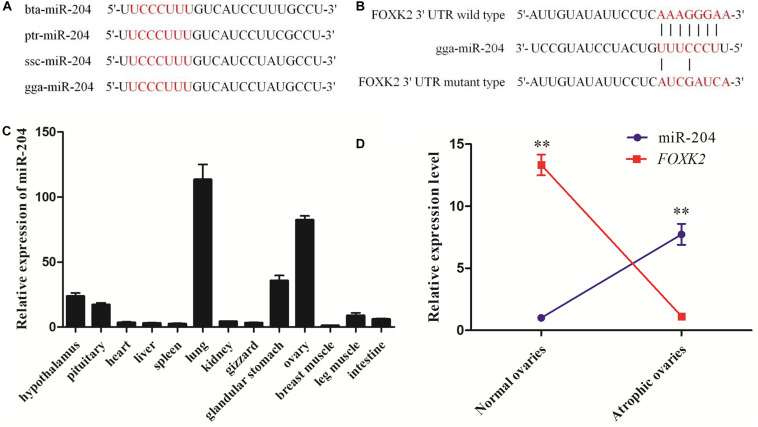
Sequencing characteristics, expression patterns, and target gene prediction of miR-204 in chicken. **(A)** The mature sequence (red characters) of miR-204 among different animal species: gga, chicken; bta, cattle; ptr, chimpanzee; ssc, pig. **(B)** The target position of the miR-204 seed sequence on the gene Forkhead box K2 (FOXK2)-3′-untranslated region (UTR) sequence (red characters) was predicted by TargetScan software. **(C)** The expression patterns of miR-204 in chicken tissues including hypothalamus, pituitary, heart, liver, spleen, lung, kidney, gizzard, glandular stomach, ovary, breast muscle, leg muscle, and intestine. **(D)** The expression levels of miR-204 and *FOXK2* in chicken atrophic ovaries and normal ovaries. Data are presented as mean ± standard error (SE); *n* = 5. miR, microRNA.

Whole-transcriptome sequencing analysis of atrophic ovaries in broody chickens revealed that miR-204 was expressed differentially between atrophic and normal ovaries. The Kyoto Encyclopedia of Genes and Genomes (KEGG) results of target genes revealed that the enriched pathways involved PI3K–Akt signaling, cell cycle, AMPK signaling, and apoptosis. We then found that FOXK2 and TRPM3 are two predicted target genes of miR-204 and are differentially expressed between atrophic and normal ovaries (Liu et al., 2018). After performing a sequence alignment, we found that the seed sequence of chicken miR-204 was conserved with mammals (Figure 1A). The seed region of miR-204 was complementary to the 3′UTR of the *FOXK2* gene, as determined using Targetscan software^1^ (Figure 1B). Additionally, miR-204 was relatively highly expressed in the chicken ovary (Figure 1C). The expression of miR-204 in atrophic ovaries was greater than in normal ovaries, whereas *FOXK2* had an opposite pattern of expression (Figure 1D).

### miR-204 Inhibits Chicken Granulosa Cell Proliferation

**FIGURE 2:**
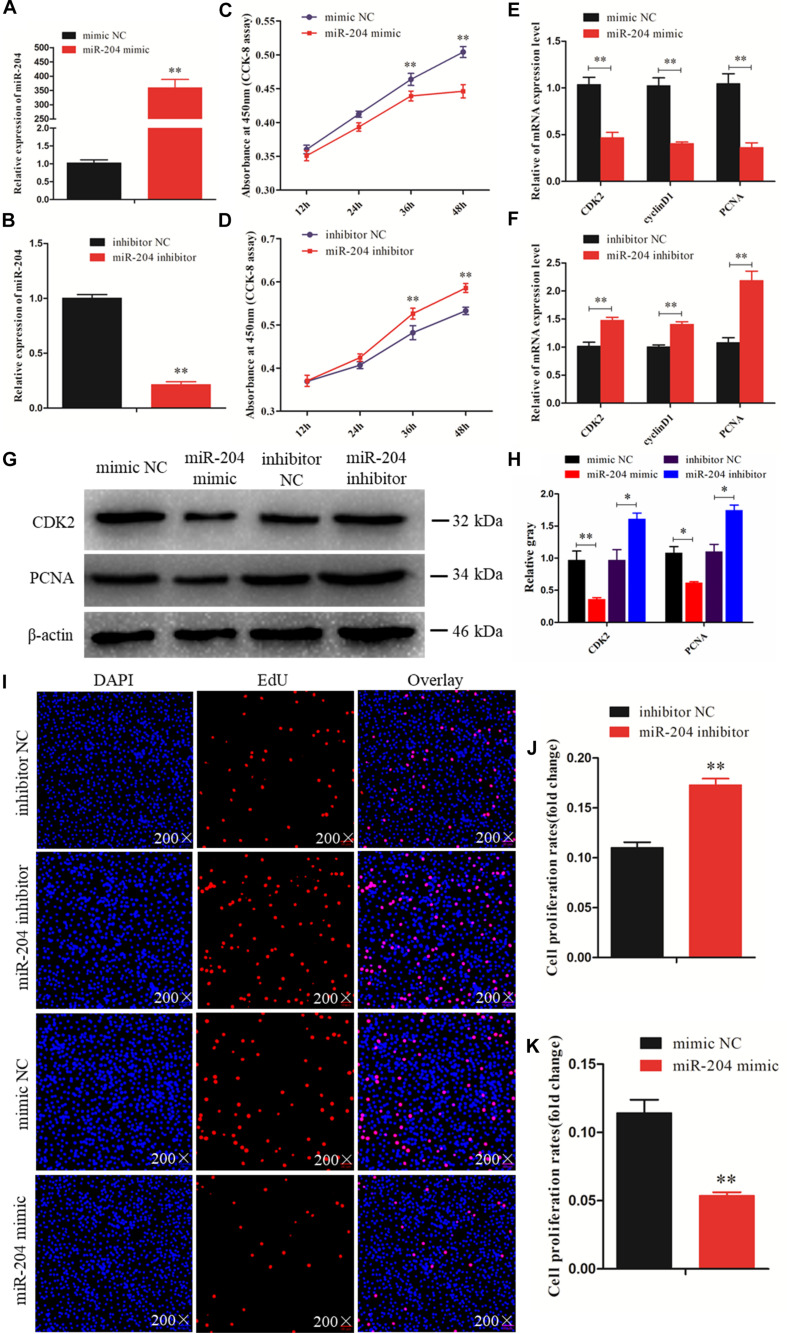
miR-204 regulates the proliferation of chicken granulosa cells (GCs). **(A)** Quantitative real-time PCR (qRT-PCR) was used to determine the miR-204 expression level after transfection of miR-204 overexpression plasmid. **(B)** The miR-204 expression level after transfection of miR-204 inhibition plasmid. **(C)** Cell proliferation curves of chicken GCs were measured with cell counting kit-8 (CCK-8) reagent after overexpression of miR-204. **(D)** Cell proliferation curves of chicken GCs were measured with CCK-8 reagent after inhibition of miR-204. **(E)** Expression abundances of cell proliferation-related genes (*PCNA*, *CDK2*, and *cyclinD1*) were detected by qRT-PCR after overexpression of miR-204. **(F)** Expression levels of cell proliferation-related genes inhibited by miR-204. **(G, H)** The protein expression levels of cell proliferation-related genes [cyclin-dependent kinase 2 (CDK2) and proliferating cell nuclear antigen (PCNA)] were detected by Western blot analysis after a gain or loss of miR-204. β-actin was used as a reference gene. **(I)** 5-Ethynyl-2-deoxyuridine (EdU) staining-positive GCs were detected by EdU kit after overexpression and inhibition of miR-204. **(J, K)** The fold change of GC proliferation rates after overexpression and inhibition of miR-204, respectively. EdU (red), 4′,6-diamidino-2-phenylindole (DAPI) (blue); Replications = 3. Data are presented as mean ± SE; *P < 0.05 and **P < 0.01. miR, microRNA; NC, negative control.

To investigate the function of miR-204 in chicken GC proliferation, a miR-204 overexpression plasmid was transfected into the GCs and its expression level increased (Figure 2A), which led to a decrease in the mRNA levels of proliferation-related genes (*CDK2*, *cyclinD1*, and *PCNA*) (Figure 2E) and protein abundance of CDK2 and PCNA (Figures 2G,H). CCK-8 reagent was used to measure the proliferative state of GCs, and the OD value was significantly decreased after transfection of a miR-204 mimic (Figure 2C). In addition, we determined the change in cell numbers by EdU staining. The results indicated that the quantities of EdU-positive cells were reduced in the miR-204 mimic group (Figures 2I,K). After transfection of a miR-204 inhibitor, there was a significant decrease in the levels of miR-204 expression (Figure 2B), while the mRNA expression of *CDK2*, *cyclinD1*, and *PCNA* increased significantly (Figure 2F). However, the protein expressions of PCNA and CDK2 were similar (Figure 2G). The CCK-8 assay results showed that there was greater proliferation in response to transfection with the miR-204 inhibitor than the NC inhibitor (Figure 2D).

The EdU assay revealed an increase in the number of proliferating cells in response to transfection with a miR-204 inhibitor (Figures 2I,J). Overall, these results demonstrate that miR-204 inhibits chicken GC proliferation.

### miR-204 Promotes Chicken Granulosa Cell Apoptosis

**FIGURE 3:**
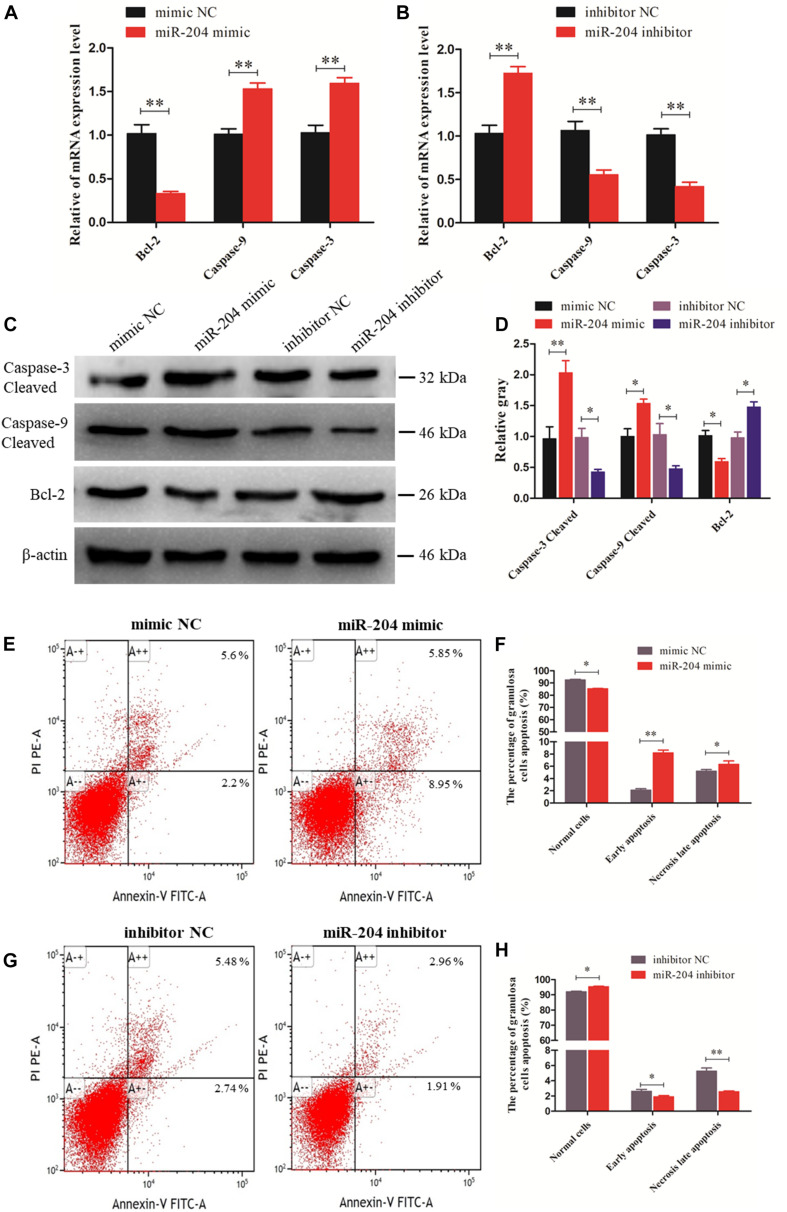
miR-204 regulates the apoptosis of chicken granulosa cells (GCs). **(A, B)** Expression abundances of cell apoptosis-related genes (*Bcl-2*, *Caspase-9*, and *Caspase-3*) were detected by quantitative real-time PCR (qRT-PCR) after overexpression and inhibition of miR-204, respectively. **(C, D)** The Western blot analysis revealed caspase-3 and caspase-9 cleavage and protein expression of Bcl-2 after a gain or loss of miR-204. β-actin was used as a reference gene. **(E)** Apoptotic GCs were detected by annexin V-fluorescein isothiocyanate (FITC)/propidium iodide (PI) staining flow cytometry after overexpression of miR-204. **(F)** The percentages of GC apoptosis were measured after overexpression of miR-204. **(G)** Apoptotic GCs were detected by annexin V-FITC/PI staining flow cytometry being inhibited by miR-204. **(H)** The percentages of GC apoptosis were determined after inhibition of miR-204. Replications = 3. Data are presented as mean ± SE; *P < 0.05 and **P < 0.01. NC, negative control.

We investigated the effect of miR-204 on chicken GC apoptosis. We found that apoptosis was promoted by transfecting a miR-204 mimic, which was associated with an increase in the mRNA and cleavage levels of caspase-9 and caspase-3 but decreased expression of Bcl-2 (Figures 3A,C,D). The miR-204 inhibitor-treated group showed a different expression trend, where there was a reduction in the expression of caspase-9 and caspase-3 but increase in Bcl-2 expression (Figures 3B–D). Flow cytometry revealed an increase in the numbers of apoptotic cells in the miR-204 mimic group compared to the mimic NC (Figures 3E,F), whereas the increased apoptotic cell numbers in the miR-204 inhibitor group eventually decreased slightly after miR-204 inhibitor transfection (Figures 3G,H). Thus, these results collectively indicate that miR-204 promotes apoptosis in chicken GCs.

### miR-204 Targets the FOXK2 Gene

**FIGURE 4:**
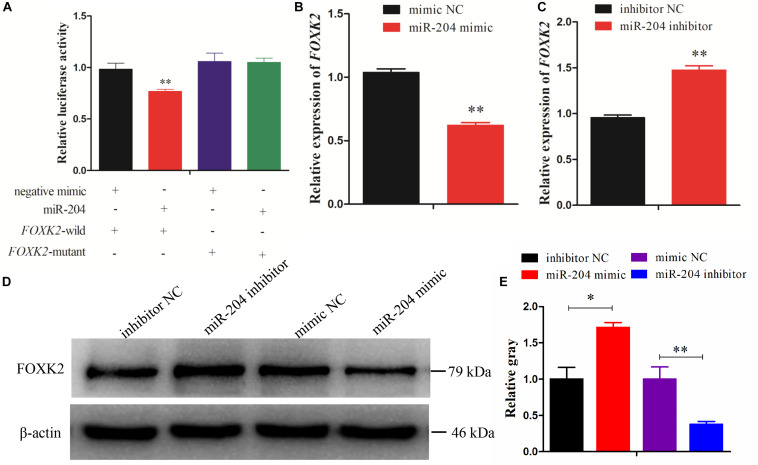
Forkhead box K2 (FOXK2) is a target gene of miR-204. **(A)** Chicken DF-1 cells were co-transfected with FOXK2-3′-untranslated region (UTR) wild or mutant dual-luciferase vector and the miR-204 mimic or miR-negative control (NC). The relative luciferase activity was assayed 48 h later. **(B, C)** The mRNA expression of FOXK2 in chicken granulosa cells (GCs) was detected by quantitative real-time PCR (qRT-PCR) after overexpression and inhibition of miR-204. **(D, E)** The protein expression of FOXK2 in chicken GCs was detected by Western blot analysis after a gain or loss of miR-204. Replications = 3. Data are presented as mean ± SE; *P < 0.05 and **P < 0.01. miR, microRNA.

Dual-luciferase reporter gene assays were performed to ascertain the direct target relationship between miR-204 and *FOXK2*. We found that the luciferase activity value of the *FOXK2* wild-type plasmid in the miR-204 group was decreased compared with the mutant-type plasmid (Figure 4A). We further analyzed the mRNA and protein expression of FOXK2 in the GCs of chickens after transfection of a miR-204 mimic or miR-204 inhibitor. The results show that there was decreased mRNA (Figure 4B) and protein (Figures 4D,E) expression of FOXK2 in the miR-204 mimic group. However, the group transfected with miR-204 inhibitor showed a significant increase in the mRNA and protein expression levels of FOXK2 (Figures 4C–E). These data demonstrate that FOXK2 is another target gene of miR-204 in chickens.

### FOXK2 Promotes Granulosa Cell Proliferation *via* Another PI3K/AKT/mTOR Regulation Pathway

**FIGURE 5:**
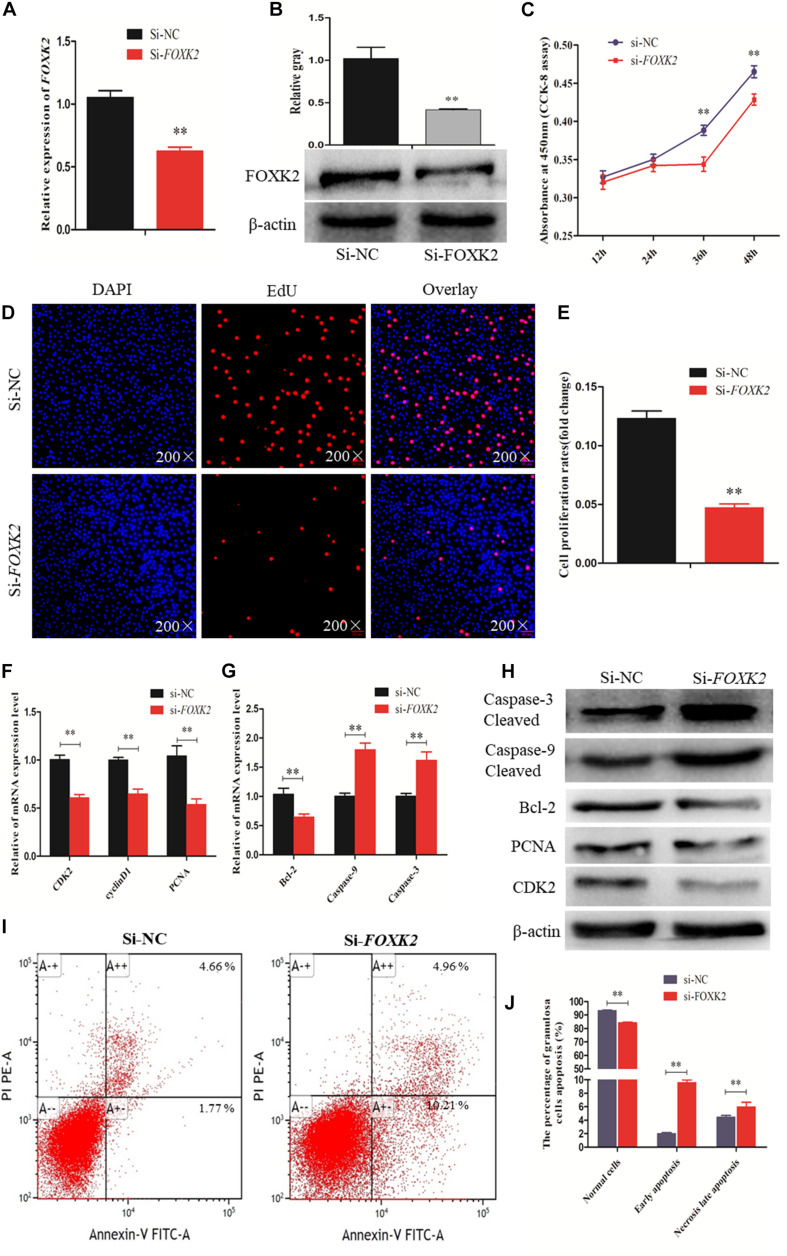
Forkhead box K2 (FOXK2) regulates chicken granulosa cell (GC) proliferation and apoptosis. **(A, B)** The mRNA and protein expressions of FOXK2 were detected after transfection of Si-FOXK2, respectively. **(C)** Cell proliferation status was detected at 450 nm with cell counting kit-8 (CCK-8) reagent after silencing FOXK2. **(D, E)** 5-Ethynyl-2-deoxyuridine (EdU) staining-positive GCs were detected by EdU kit after downregulation of FOXK2. **(F)** The mRNA expression levels of cell proliferation-related genes (*PCNA*, *CDK2*, and *cyclinD1*) were detected by quantitative real-time PCR (qRT-PCR) after FOXK2 knockdown. **(G)** The mRNA expressions of cell apoptosis-related genes (*Bcl-2*, *Caspase-9*, and *Caspase-3*) were detected by qRT-PCR after downregulation of FOXK2. **(H)** The protein expression levels of cell proliferation-related genes (CDK2 and PCNA) and cell apoptosis-related gene (Bcl-2, Caspase-9, and Caspase-3) were detected by Western blot analysis after FOXK2 knockdown. **(I, J)** Apoptotic GCs were detected by annexin V-fluorescein isothiocyanate (FITC)/propidium iodide (PI) staining flow cytometry following FOXK2 silencing. Replications = 3. Data are presented as mean ± SE; Si, small RNA interference. *P < 0.05 and **P < 0.01.

A recent study linked FOXK2 to regulation of liver cellular proliferation and apoptosis *via* the PI3K–Akt pathway (Sakaguchi et al., 2019). In this study, we examined the role of FOXK2 knockdown on chicken GC proliferation and apoptosis. Initially, the mRNA and protein expression of FOXK2 was significantly inhibited after treatment with FOXK2-specific siRNA (Figures 5A,B). Then, cells in a proliferation state were identified by CCK-8 and EdU staining, and the numbers of apoptotic cells were determined using flow cytometry. The results showed that Si-*FOXK2* influenced the decline in the OD value and EdU-positive cell numbers in the GCs (Figures 5C–E), while the numbers of early apoptotic cells were higher than in Si-NC group (Figures 5I,J). Finally, we detected changes in the expression of proliferation-related genes and proapoptotic factors. The results showed that the levels of mRNA and protein expression of caspase-3 and caspase-9 increased significantly, whereas the expression of Bcl-2, PCNA, and CDK2 decreased (Figures 5F–H). Thus, FOXK2 promotes the proliferation and inhibits apoptosis of chicken GCs.

**FIGURE 6:**
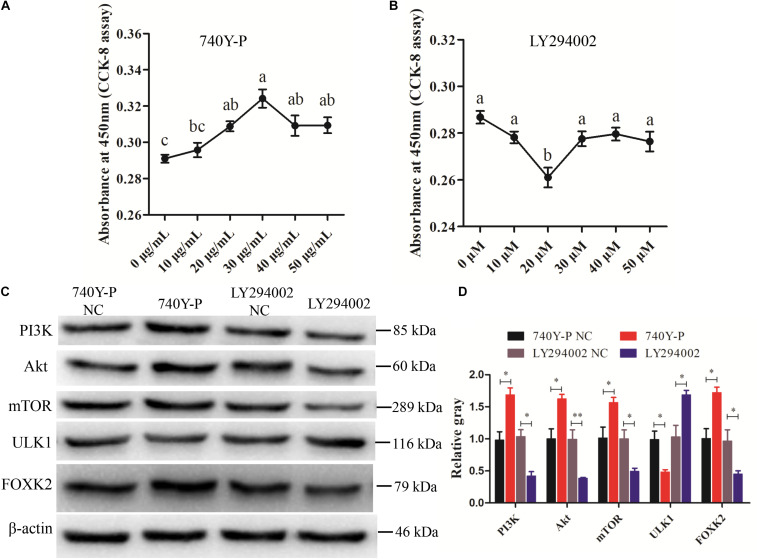
Phosphoinositide 3-kinase (PI3K)/AKT/mammalian target of rapamycin (mTOR) pathway analysis. **(A, B)** Cell counting kit-8 (CCK-8) assay method was employed to determine the optimum concentration of PI3K activator (740Y-P) and PI3K inhibitor (LY294002) treatments. **(C, D)** The protein expressions of PI3K/AKT/mTOR pathway (PI3K, AKT, and mTOR) and downstream regulators [Forkhead box K2 (FOXK2) and ULK1] were detected by Western blot after the treatments of 740Y-P and LY294002. β-actin was used as a reference gene. Replications = 3. Data are presented as mean ± SE; *P < 0.05 and **P < 0.01.

Previous research reported that FoxKs translocation to the nucleus is dependent on the PI3K–Akt–mTOR pathway (Sakaguchi et al., 2019). To further explore the role of FOXK2 in the PI3K signaling pathway, a PI3K inhibitor (LY294002) and activator (740Y-P) were used. The optimal concentrations of 740Y-P and LY294002 were tested and found to be 30 μg/ml and 20 μM, respectively (Figures 6A,B). The results showed that 740Y-P significantly increased the protein expression of PI3K. Simultaneously, the protein expressions of Akt and mTOR were higher than those in the 740Y-P NC group, which resulted in an increased expression of FOXK2. On the contrary, LY294002 inhibited PI3K and also decreased the protein expression of Akt and mTOR, which led to a reduction in the protein expression of FOXK2 (Figures 6C,D). Meanwhile, the protein expression of ULK1 in the 740Y-P group was inhibited, while it was promoted in the LY294002 group. These results revealed that both FOXK2 and ULK1 are downstream regulators of PI3K/Akt/mTOR signal pathways and might be related to cell proliferation and autophagy.

### miR-204 Regulates Granulosa Cell Autophagy by Targeting TRPM3

**FIGURE 7:**
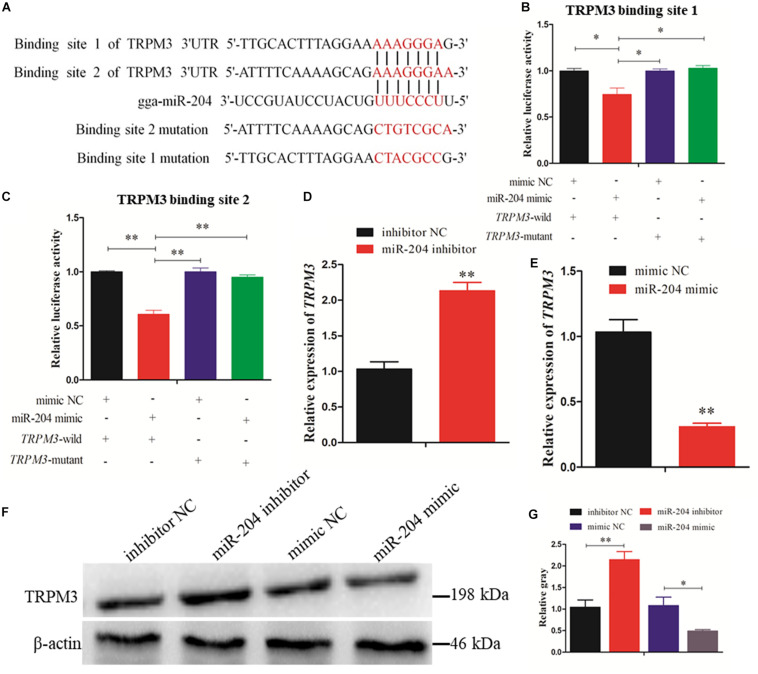
Dual-luciferase reporter gene assays confirmed that miR-204 directly interacts with these two binding sites of transient receptor potential melastatin 3 (TRPM3). **(A)** The target positions of miR-204 seed sequence on TRPM3 and wild or mutant sequence of TRPM3-3′-untranslated region (UTR). **(B, C)** Chicken DF-1 cells were co-transfected with TRPM3-3′-UTR wild or mutant dual-luciferase vector and the miR-204 mimic or mimic negative control (NC). The relative luciferase activity was assayed 48 h later. **(D, E)** The mRNA expression of TRPM3 was detected after overexpression or inhibition of miR-204. **(F, G)** The protein levels of TRPM3 in chicken granulosa cells (GCs) were detected after transfection of overexpression or inhibition of miR-204. β-actin was used as a reference gene. Replications = 3. Data are presented as mean ± SE; *P < 0.05 and **P < 0.01.

Two predicted miR-204 binding sites in the TRPM3 3′UTR were identified by TargetScan (Figure 7A). Dual-luciferase reporter gene assays were used to validate whether miR-204 can directly interact with these two binding sites of TRPM3. The results showed that the luciferase activities of the two binding sites were decreased in response to miR-204 mimic (Figures 7B,C). Furthermore, both mRNA and protein expression levels of TRPM3 were increased by miR-204 inhibition (Figures 7D,F,G), while overexpression of miR-204 inhibited mRNA and protein expression of TRPM3 (Figures 7E–G). The results thus demonstrate that TRPM3 is another target gene of miR-204.

**FIGURE 8:**
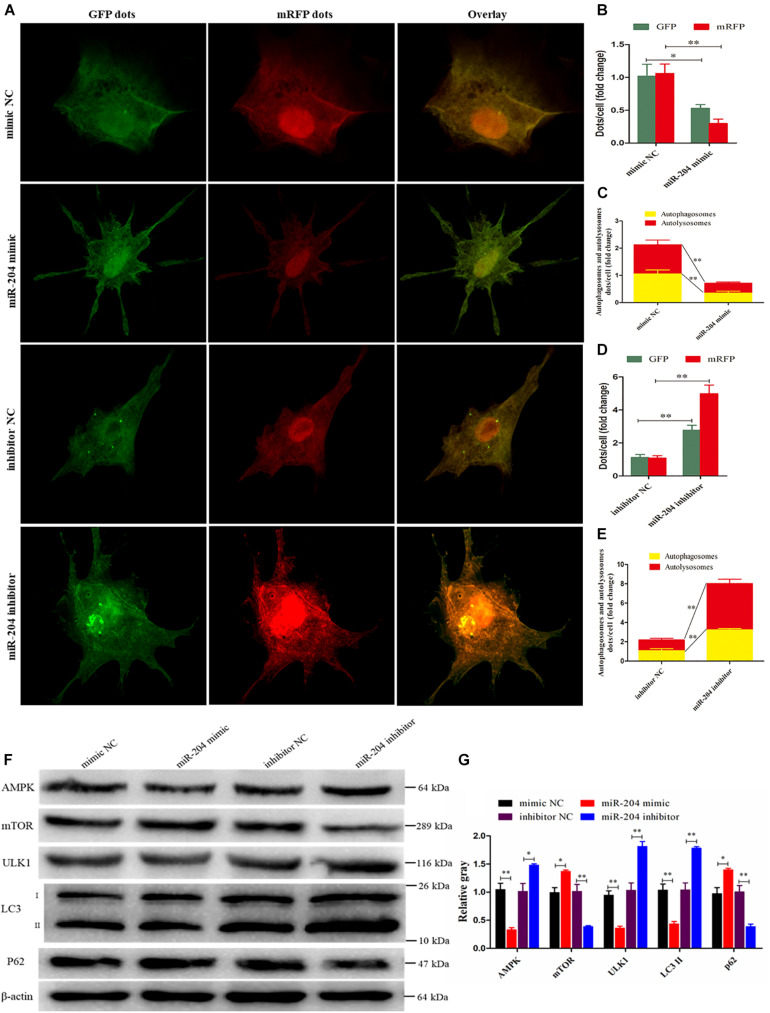
miR-204 regulates granulosa cell (GC) autophagy *via* transient receptor potential melastatin 3 (TRPM3)/AMP-activated protein kinase (AMPK)/ULK1 pathway. **(A)** An adenovirus harboring tandem fluorescent mRFP-GFP-LC3 was used to evaluate the extent of autophagic flux after transfection of overexpression or inhibition of miR-204. **(B, D)** Mean number of green fluorescent protein (GFP) and monomeric red fluorescent protein (mRFP) dots per cell; nine cells were randomly selected to count for each field. **(C, E)** Mean number of autophagosomes and autolysosomes per cell. The autophagosomes were dotted with both green (GFP) and red (mRFP) color, and overlaid images showed with a yellow color. Autolysosomes were dotted with mRFP but not GFP, and overlaid images showed with a red color. **(F, G)** The protein levels of TRPM3/AMPK/ULK1 pathway [AMPK, mammalian target of rapamycin (mTOR), ULK, LC3-II, and p62] were detected after transfection of overexpression or inhibition of miR-204. β-actin was used as a reference gene. Replications = 3. Data are presented as mean ± SE; *P < 0.05 and **P < 0.01.

Subsequent experiments were performed to evaluate the extent of autophagic flux using an adenovirus harboring tandem fluorescent mRFP-GFP-LC3, which differentiates between autophagosomes and autolysosomes (Kimura et al., 2007). The autophagosomes were dotted with both green [green fluorescent protein (GFP)] and red [monomeric red fluorescent protein (mRFP)] colors, and overlaid images revealed a yellow color where there was co-localization. Autolysosomes were dotted with mRFP but not GFP, and overlaid images showed a red color (Hariharan et al., 2011). After transfecting a miR-204 mimic, the dot numbers of GFP and mRFP were decreased (Figures 8A,B). In the overlaid images, fewer red dots were observed, indicating decreased autolysosome synthesis (Figures 8A,B). Compared with the NC inhibitor, the increased red dots reflected an increased level of autophagic flux after transfection of the miR-204 inhibitor (Figures 8A,D,E). These data suggest that miR-204 inhibits GC autophagy by suppressing TRPM3.

### miR-204 Impedes the TRPM3/AMPK/ULK1 Pathway

miR-204 and TRPM3 regulate autophagy through the AMPK/ULK1 pathway (Hall et al., 2014). After transfection of a miR-204 mimic, the protein expressions of TRPM3, AMPK, and ULK were decreased, while mTOR protein expression was increased. Notably, after the overexpression of miR-204, the expression of LC3-II was significantly reduced, whereas p62, a polyubiquitin-binding protein known to be degraded during autophagy (Bjorkoy et al., 2005; Pankiv et al., 2007), was significantly increased. In contrast, TRPM3, AMPK, and ULK protein levels were increased, and mTOR protein expression was reduced after the knockdown of miR-204. miR-204 inhibition promotes the accumulation and degradation of LC3-II and p62, respectively (Figures 8F,G), which enhanced autophagy. These results confirmed that miR-204 inhibits autophagy by impeding the TRPM3/AMPK/ULK pathway.

### TRPM3 Promotes Granulosa Cell Autophagy

**FIGURE 9:**
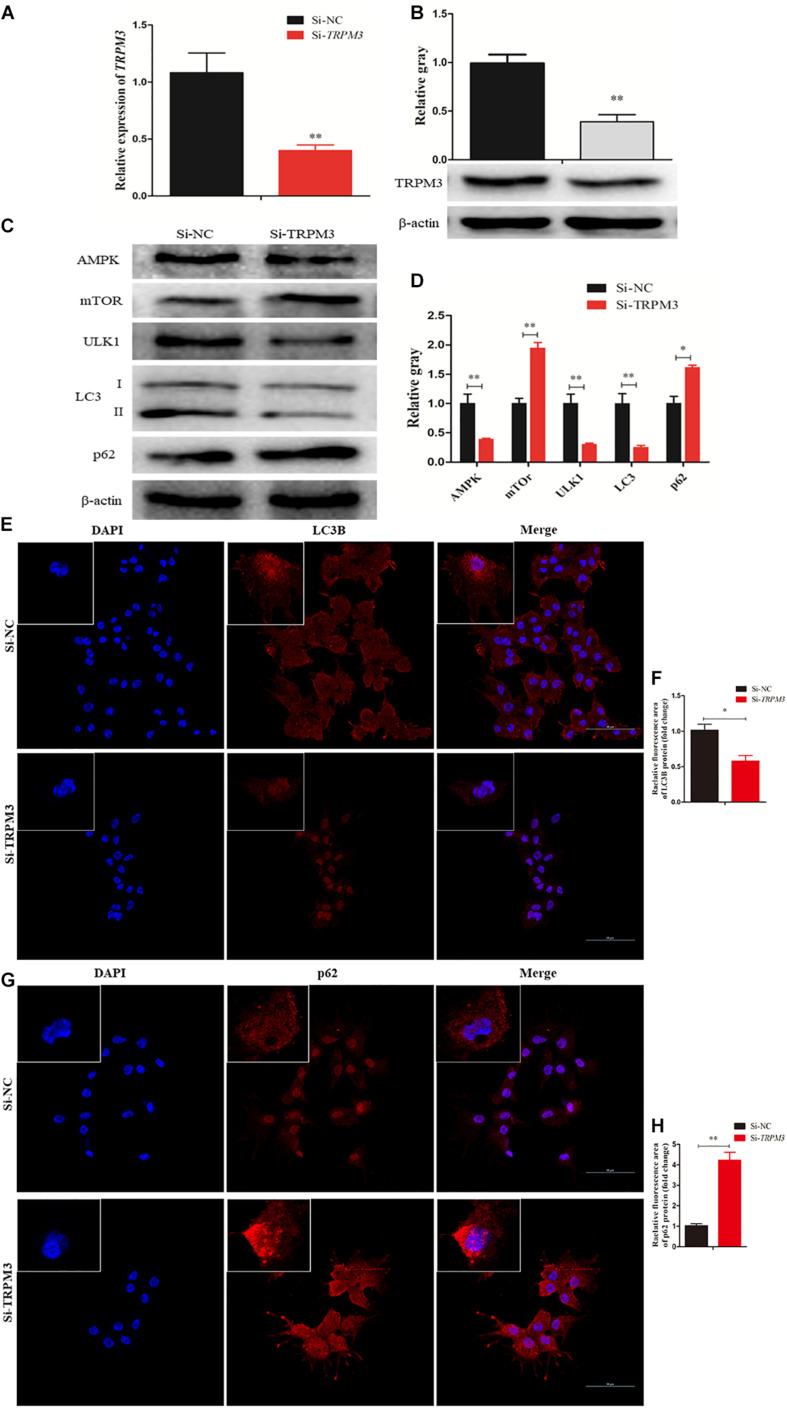
Transient receptor potential melastatin 3 (TRPM3) promotes granulosa cell (GC) autophagy. **(A, B)** The mRNA and protein expressions of TRPM3 were detected after transfection of Si-TRPM3. **(C, D)** The protein levels of TRPM3/AMP-activated protein kinase (AMPK)/ULK1 pathway [AMPK, mammalian target of rapamycin (mTOR), ULK, LC3-II, and p62] were detected after transfection of Si-TRPM3. β-actin was used as a reference gene. **(E, F)** Immunofluorescence analysis was performed to test the fluorescence intensity of LC3B after TRPM3 silencing. **(G, H)** Immunofluorescence analysis was performed to test the fluorescence intensity of p62 after TRPM3 silencing. Replications = 3. Data are presented as mean ± SE; *P < 0.05 and **P < 0.01.

Recent studies have demonstrated that TRPM3 is involved in the regulation of autophagy (Hall et al., 2014; Cost and Czyzykkrzeska, 2015). Therefore, we investigated the effect of TRPM3 knockdown on GC autophagy using siRNA. The results showed that the expression of TRPM3 was significantly inhibited (Figures 9A,B), and the protein expressions of AMPK and ULK1 were reduced, while the expressions of mTOR and p62 increased significantly. TRPM3 knockdown impeded the accumulation of LC3-II as compared with the Si-NC group (Figures 9C,D). Immunofluorescence showed that the fluorescent intensity of LC3B reduced significantly (Figures 9E,F), whereas p62 protein presented an opposite pattern after TRPM3 silencing (Figures 9G,H). Based on these results, we suggest that TRPM3 promotes autophagy in GCs. Thus, miR-204 is associated with chicken GC apoptosis and autophagy *in vivo*.

## Discussion

Ovarian tumors are associated with a high risk of morbidity and mortality (Wootipoom et al., 2006; Yamagami et al., 2017). Atrophic ovary is a common type of ovarian tumor accompanied by an increase in follicular atresia and GC apoptosis and autophagy (Fredrickson, 1987; Manabe et al., 2002; Yang et al., 2017). Our previous research showed that miR-204 was differentially expressed between chicken atrophic ovaries and normal ovaries (Liu et al., 2018). Aberrant expression of miR-204 has been frequently reported in cancer studies and correlates with cell proliferation and autophagy (Jian et al., 2011; Liu et al., 2013; Zhou et al., 2014; Wu et al., 2015). In this study, we speculated that miR-204 plays an important role in chicken GC proliferation, apoptosis, and autophagy. Therefore, series of experiments were conducted, and the results showed that miR-204 impeded chicken GC proliferation and promoted apoptosis by targeting *FOXK2*.

FOXK2 is a member of the Foxk family of forkhead transcription factors, which is specifically involved in regulating the balance between proliferation, differentiation, and cell survival (Der Heide et al., 2015). Previous studies demonstrated that FOXK2 suppressed the growth of lung cancer cells by targeting cyclin D1 and CDK4 (Chen et al., 2017) and also influenced CDKs, which linked it to the regulation of the cell cycle (Marais et al., 2010). Additionally, FOXK2 was reported to be involved in cellular processes and important signaling pathways, such as the p53 pathway (Shan et al., 2016), Wnt/β-catenin pathway (Wang et al., 2015), and the PI3K–Akt pathway (Lin et al., 2017). Overexpression of FOXK2 enhanced hepatocellular growth, whereas FOXK2 suppression is associated with decreased cell survival (Lin et al., 2017). Our current study showed that overexpression of miR-204 promoted GC apoptosis, which was characterized by increasing apoptotic cell numbers and gene expression of caspase-9 and caspase-3. Meanwhile, CCK-8 and EdU results indicated that a miR-204 mimic had low cell vitality and numbers of EdU-positive cells, and the gene expression of PCNA, CDK2, and cyclinD1 was decreased. Subsequently, the bioinformatics analyses and dual-luciferase reporter gene assays were performed and demonstrated that *FOXK2* is directly targeted by miR-204. Both mRNA and protein expressions of FOXK2 were suppressed by miR-204, and further functional experiments indicated that *FOXK2* had the opposite effect of miR-204 on GC proliferation and apoptosis. After *FOXK2* knockdown, we found that proliferation of GCs was inhibited and the numbers of apoptotic cells were increased. A similar result was reported in liver cells (Sakaguchi et al., 2019).

**FIGURE 10:**
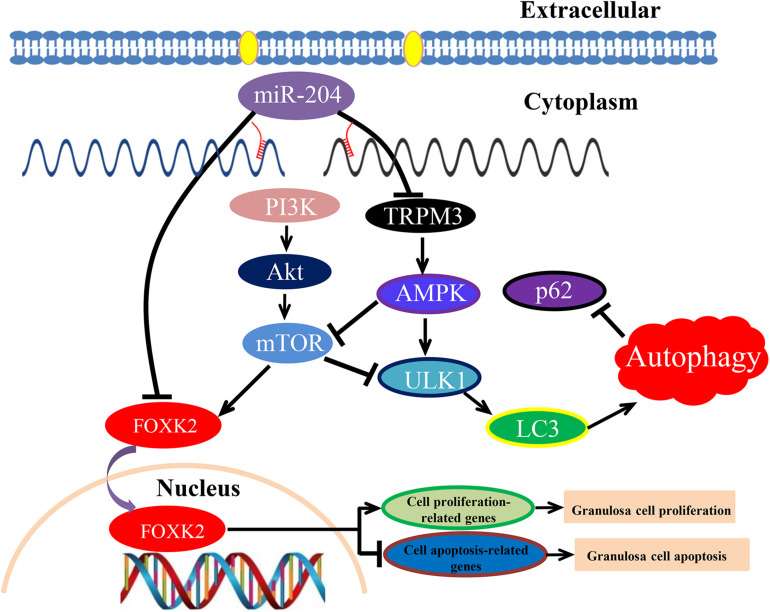
Schematic model of miR-204-mediated regulatory mechanism in chicken granulosa cell (GC) proliferation, apoptosis, and autophagy. miR-204 promotes GC apoptosis by repressing Forkhead box K2 (FOXK2) *via* the phosphoinositide 3-kinase (PI3K)/AKT/mammalian target of rapamycin (mTOR) regulation pathway and inhibits autophagy by impeding the transient receptor potential melastatin 3 (TRPM3)/AMP-activated protein kinase (AMPK)/ULK pathway.

The PI3K/AKT/mTOR signaling pathway contributes to a variety of processes that are critical in mediating many aspects of cellular function, including cell proliferation and survival, gene expression, and metabolic activities (Yu and Cui, 2016). PI3K plays a key role in regulating cell proliferation, which is indispensable for cell self-renewal (Mclean et al., 2007; Zhou et al., 2011). Akt is an important downstream target in the PI3K signal transduction pathway, which can promote cell survival and inhibit apoptosis (Tu et al., 2018). As a vital activator for Akt, mTOR plays a critical role in cell survival and differentiation (Sugatani and Hruska, 2005; Rao et al., 2010). PI3K/Akt/mTOR signaling regulated GC proliferation and apoptosis by targeting its downstream protein FOXK2 (Figure 10).

Increased expression of cleaved caspase-3 and caspase-9 led to the inhibition of autophagy and enhanced apoptosis (Xiong et al., 2015). We found that miR-204 regulated GC autophagy by targeting TRPM3. Autophagy is a complex cellular process that is essential for cell homeostasis (Stromhaug and Klionsky, 2001); therefore, in either extreme or less-extreme autophagy, cell death can occur (Sadoshima, 2008; Hall et al., 2014). Autophagy is regulated by many autophagy-related genes (Atgs), which are involved in autophagosome formation (Nishida et al., 2009). LC3 (microtubule-associated protein 1 light chain 3, Atg8) is essential for the formation of an autophagosome (Kabeya et al., 2000). The soluble form of LC3 (LC3-I), which transforms into the autophagic vesicle-associated form (LC3-II), is an important marker of effective autophagy (Asanuma et al., 2003). ULK1 (unc-51 like autophagy activating kinase 1, Atg1) is an autophagy-initiating kinase; therefore, it initiates autophagy (Shang and Wang, 2011).

Recently, it was established that miR-204 directly inhibits the translation of TRPM3, and a loss of miR-204 leads to a high expression of TRPM3 and stimulates autophagy (Cost and Czyzykkrzeska, 2015). Similarly, Hall et al. (2014) demonstrated that miR-204 acts as a critical regulator of autophagy by targeting TRPM3, and overexpression of TRPM3 leads to activation of AMPK and ULK1, which forms a connection with the autophagic pathway (Hall et al., 2014). In our study, we confirmed that TRPM3 is another target gene of miR-204 and regulates GC autophagy by targeting the AMPK/ULK1 pathway. AMPK and mTOR (target of rapamycin) are upstream regulators of ULK1. AMPK is involved in promoting autophagy by directly activating ULK1, while the activity of mTOR prevents the activation of ULK1 and also disrupts the interaction between ULK1 and AMPK (Kim et al., 2011). Overexpression of miR-204 attenuated autophagic flux and reduced the protein expression of TRPM3, AMPK, ULK, and LC3-II; however, it also resulted in increased protein expression of mTOR and p62. On the contrary, miR-204 inhibition enhanced autophagic flux in cultured GCs and led to increased protein expression of TRPM3, AMPK, and ULK but decreased protein expression of mTOR. miR-204 inhibition resulted in accumulation and degradation of LC3-II and p62, indicating that miR-204 inhibited autophagy by impeding the TRPM3/AMPK/ULK pathway (Figure 10).

In conclusion, these results show that miR-204 is highly expressed in chicken atrophic ovaries and functions in promoting GC apoptosis by repressing FOXK2 *via* the PI3K/AKT/mTOR regulation pathway and inhibiting autophagy by impeding the TRPM3/AMPK/ULK pathway.

## Data Availability Statement

The raw data supporting the conclusions of this article will be made available by the authors, without undue reservation.

## Ethics Statement

The animal study was reviewed and approved by the Institutional Animal Care and Use Committee at the Sichuan Agricultural University (No. YCS-B2018102013), and all laboratory works conducted were in accordance with the Sichuan Agricultural University (SAU) Laboratory Animal Welfare and Ethics guidelines.

## Author Contributions

XZ, ZC, and LL conceived and designed the experiments. ZC, LL, and FK performed the experiments. ZC, FK, QZ, YW, DL, GS, and YT analyzed the data. ZC and XZ wrote the manuscript. All authors contributed to the article and approved the submitted version.

## Conflict of Interest

The authors declare that the research was conducted in the absence of any commercial or financial relationships that could be construed as a potential conflict of interest.

## Footnotes

1 http://www.targetscan.org/vert_72/
