# Supplementary material for: High Expression of miR-204 in Chicken Atrophic Ovaries Promotes Granulosa Cell Apoptosis and Inhibits Autophagy
Source: Front Cell Dev Biol. 2020 Nov 5;8:580072. doi: 10.3389/fcell.2020.580072 (PMC7676916; doi:10.3389/fcell.2020.580072)
Supplement: Supplementary file 1 [file Table_1.DOCX]

**Table S1. Oligonucleotide sequences used in this study**

| Name | Sequences (5'-3') |
| --- | --- |
| gga-miR-204 mimic | UUCCCUUUGUCAUCCUAUGCCU |
| gga-miR-204 inhibitor | AGGCAUAGGAUGACAAAGGGAA |
| Si-*FOXK2* | GCAGCUACCACAAACUAUUTT |
| Si-*TRPM3* | GCUCGCAGUCAGAUCUUUATT |

**Table S2. Primers used for qRT-PCR**

| Gene | Sequence (5' - 3') | Product Length (bp) | Annealing Temperature (℃) |
| --- | --- | --- | --- |
| *FOXK2* | F: CAATCGGAGCTGCTAGTCGT | 134 | 58 |
|  | R: TATGTGCGTTCCGTTTTGCG |  |  |
| *CDK2* | F: CCAGAACCTCCTCATCAAC | 171 | 60 |
|  | R: CAGATGTCCACAGCAGTC |  |  |
| *Cyclin D1* | F: CAGAAGTGCGAAGAGGAAGT | 188 | 58 |
|  | R: CTGATGGAGTTGTCGGTGTA |  |  |
| *Bcl-2* | F: ATCGTCGCCTTCTTCGAGTT | 150 | 58 |
|  | R:ATCCCATCCTCCGTTGTCCT |  |  |
| *PCNA* | F:GAGACCTCAGCCACATTGGT | 173 | 59 |
|  | R:AGTCAGCTGGACTGGCTCAT |  |  |
| *Caspase-3* | F: TGGCCCTCTTGAACTGAAAG | 106 | 61 |
|  | R: TCCACTGTCTGCTTCAATACC |  |  |
| *Caspase-9* | F: TCCCGGGCTGTTTCAACTT | 270 | 61 |
|  | R: CCTCATCTTGCAGCTTGTGC |  |  |
| *TRPM3* | F: GCAGAGCAGGCACAGACTGATTC | 99 | 59 |
|  | R: CCGATGTACGCCAGTGTGTAGAAC |  |  |
| *GAPDH* | F: TCCTCCACCTTTGATGCG | 146 | 60 |
|  | R: GTGCCTGGCTCACTCCTT |  |  |
